# Susceptible Antiferroelectric/Ferroelectric Transitions in Silver Niobate-Based Ceramics Induced by Manual Mechanical Processing

**DOI:** 10.1021/acs.inorgchem.5c03344

**Published:** 2026-02-04

**Authors:** Ye Tian, Teng Lu, Shaoqi Guo, Chen Chen, Liaona She, Li Jin, Ray L. Withers, Yuanting Wu, Wanyin Ge, Xiaoyong Wei, Haixue Yan, Yun Liu

**Affiliations:** † School of Materials Science and Engineering, Shaanxi University of Science & Technology, Xi’an, Shaanxi Province 710021, P. R. China; ‡ Electronic Materials Research Laboratory, School of Electronic and Information Engineering, Xi’an Jiaotong University, Xian, Shaanxi Province 710049, China; § Research School of Chemistry, The Australian National University, Canberra, ACT 2601, Australia; ∥ State Key Laboratory of High Performance Ceramics and Superfine Microstructure, 58306 Shanghai Institute of Ceramics Chinese Academy of Sciences, Shanghai 200050, China; ⊥ Institute of Science and Technology for New Energy, Xi’an Technological University, Xi’an, Shaanxi Province 710021, P. R. China; # School of Engineering and Materials Science, Queen Mary University of London, Mile End Road, London E1 4NS, U.K.

## Abstract

It is common that the as-sintered bulk ceramics or powders undergo mechanical and/or heat treatment for further investigating the structure or properties. However, if their structures change during these procedures, it would mislead their structure–properties relation and subsequently lead to the wrong design of the desired ceramic products. In this work, we reported the mechanical/heat treatment effect on the structures of AgNbO_3_-based antiferroelectric/ferroelectric (AFE/FE) materials. The results revealed that the structure of AgNbO_3_-based systems with chemical compositions close to/in the AFE/FE phase boundary strongly depends on the history of polishing/grinding and/or annealing processing. Manual polishing/grinding (or milling) ceramic bulk/powders can induce a phase transition from AFE­(*Pbcm*) to FE­(*R*3*c*), and the transition can be reversed via the heat treatment, which is intimately related to a temperature-driven phase transformation having a metastable AFE phase. Our finding thus provides new insight into phase transitions in AgNbO_3_-based systems, suggesting that the proper parameters of processing need to be considered to enable the development of an understanding of structure–property relationships in ceramic materials.

## Graphical abstract

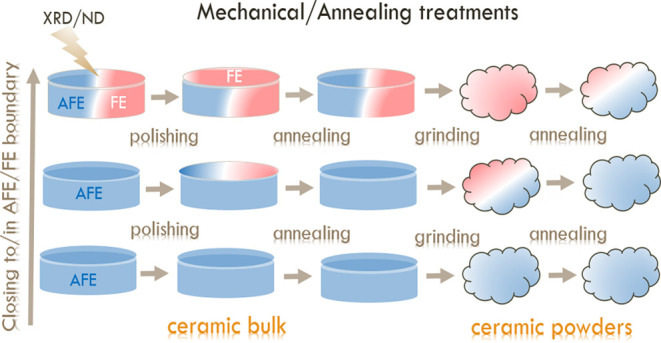

## 1 Introduction

Anti/ferroelectric (AFE/FE) perovskites play a crucial role in the development of modern electronics and associated smart electronic devices.^1–4^ They have been successfully utilized to fabricate actuators, transducers, sensors, pulse high-power capacitors, solid-state cooling devices, and infrared detectors based on their high piezoelectric, electrostrain, polarization, electrocaloric, and pyroelectric properties.^5–18^ However, the most commercialized AFE/FE materials are lead-containing compounds, e.g., lead zirconate titanate (PZT). Considering the issues caused to the environment and human health, lead-free alternatives are being sought.^5,10,11,19–25^

In the lead-free perovskite family, silver niobate (AgNbO_3_) was initially recognized as a weak-FE, with a tiny polarization hysteresis observed under a weak applied field (17 kV cm^–1^).^26^ The structure studies together with property measurement under an ultrahigh applied field (220 kV cm^–1^), confirmed its AFE property by observing antiparallel Ag^+^ and Nb^5+^ displacements in a centrosymmetric nonpolar structure and classic double polarization–electric field (P–E) hysteresis.^27–29^ Recently, it was discovered that AgNbO_3_ exhibited a ferrielectric (i.e., noncompensated AFE) structure modeling in a noncentrosymmetric polar structure.^30–32^ Theoretical calculations also suggested that such a model reveals the coexistence of FE and AFE phases with very similar energy states (the difference only 0.1 meV/fu) to understand its anti/ferroelectric nature.^33^ Although the exact crystallographic symmetry of AgNbO_3_ is controversial to date since its discovery in 1958, enhancing its anti/ferroelectric property is increasingly attracting the interest of scientists and engineers globally.^34–37^ It was reported that the anti- or ferroelectricity of AgNbO_3_ can be stabilized or even enhanced via a chemical approach. For example, enhanced ferroelectricity can be achieved in Li^+^- or K^+^-doped AgNbO_3_,^38–41^ while doping with Bi^3+^, rare earth (La^3+^, Sm^3+^, etc.), and/or Ta^5+^ can promote the stability of its antiferroelectricity.^37,42–52^

The structural stability of AFE or FE assisted by element doping resulted in the chemically modified AgNbO_3_-based perovskite ceramics showing great potential use in microwave, photovoltaics/photocatalysis, piezoelectric/force electrics, pyroelectrics, and energy storage devices.^44,53–67^ However, there are rare studies on their structure–property relation, especially the chemical compositions close to or in the AFE/FE phase boundary. For electroceramic products, it is well known that bulk samples are usually adopted to characterize the electrical properties, while the samples used to collect structure information via the X-ray diffraction (XRD) method are, in most cases, in the form of ceramic powders. Furthermore, an X-ray beam can only penetrate a few microns depth of the ceramic bulk, which can only collect the structure information on the near-surface regions of the ceramic bulk. Mechanical polishing/grinding (or milling) and annealing processes are common procedures to prepare samples for structure or property characterization. If these predesigned procedures induce a structural change, the structure information obtained from XRD would not reflect the real structure of the original samples, which will lead to the misunderstanding of the structure–property relationship and subsequently result in wrong product design. Our previous publication reported a polishing-induced FE/AFE phase transition on a Pb-based FE ceramic bulk, revealing such a structural instability near the AFE/FE phase boundary.^68^ To further clarify whether such a characteristic has universality, in this work, we symmetrically investigated the manual mechanical/heat treatment effects on the structure of (1–*x*)­AgNbO_3_–*x*LiTaO_3_ (ANLT*x*, *x* = 4.5, 5.3, 6 mol %) solid solution system (including their form of ceramic bulk and powders), where these chemical compositions were reported near/in the AFE/FE phase boundary in previous publications.^36,58^ Additionally, to confirm whether the manual mechanical/heat treatment effect can also result in structural change with the chemical compositions outside the AFE/FE phase boundary, we also compare the XRD raw data of ANLT*x* (*x* = 0, 1.5, 3, 3.8 mol %).

## 2 Experimental Procedure

Polycrystalline ceramic samples were synthesized by a conventional solid-state process using a standard mixed oxide route. Ag_2_O (99.7%), Nb_2_O_5_ (99.99%), Li_2_CO_3_ (99.5%), and Ta_2_O_5_ (99.99%) were weighed according to the nominal formula as (1–*x*)­AgNbO_3_-*x*LiTaO_3_ (ANLT*x*, *x* = 0, 1.5, 3, 3.8, 4.5, 5.3, 6 mol %) and ball-milled in ethanol for 12 h. After drying, the mixtures were put into alumina porcelain boats and calcined at 850–950 °C for 4 h in an oxygen atmosphere with a heating rate of 5 °C. The calcined powders were again ball-milled for 4 h in an ethanol medium. After drying, the powders were mixed with a 5 wt % poly­(vinyl alcohol) (PVA) solution and pressed into pellets with a diameter of 15 mm and a thickness of 1∼2 mm under 400 MPa uniaxial pressure. After burning out PVA at 600 °C for 2 h, the samples were finally sintered at 1060–1120 °C for 6 h in an oxygen atmosphere, followed by cooling at a rate of 5 °C min^–1^ down to 500 °C and naturally in the furnace. The color of all of the ceramic pellets is bright yellow. Relative densities of all studied samples estimated by the Archimedes method were larger than 97%.

X-ray diffraction (XRD) patterns of both ceramic bulks and powders treated under historically different manners and conditions are collected on a PANalytical X’Pert Empyrean diffractometer fitted with an X’Celerator detector, in the Bragg–Brentano reflective geometry using Ni-filtered Cu-Ka radiation (λ = 1.5418Å), over the 2θ range of 20–100° with a step of 0.026°. The total collected time is about 2 h. Before collecting the XRD data of the ceramic bulk, the treated ceramic pellets were cleaned for 2 min in an ultrasonic cleaner. For the collection of temperature-dependent XRD data, the sample platform was connected to a computer-controlled temperature chamber. The structure refinement of ceramic powder samples with selected chemical compositions is carried out using the GSAS suite program.^69^

The processing situations of each individual sample are described as follows. The as-sintered (i.e., fresh) ceramic pellets without the polishing process are denoted as sample A series. The as-sintered ceramic pellets that are well polished using diamond abrasive paper show a mirror-like fine surface with minimum artificial scratching after polishing (i.e., sample B series). It should be noted that coarse polishing can cause a number of scratches, destroying the fine structure in the near-surface regions of ceramic bulk (evidenced by the sharp splitting characteristic of perovskite diffraction peaks that were replaced by broad diffraction peak profiles). The polished ceramic pellets are further annealed at 600 °C for 10 min, which are named as sample C series. That means those ceramic pellets with the C series experienced polishing → annealing treatment (denoted as annealed ceramic bulk) compared with the A series. The ceramic bulks are crushed and ground into fine ceramic powders, denoted as ground ceramic powders (i.e., powder B series). The ground ceramic powders are further annealed at elevated temperatures. In particular, the 600 °C annealed ceramic powders are denoted as powder C series, which means powder C series experiences grinding → annealing processing. The 600 °C annealed ceramic powders further experience regrinding, denoted as reground ceramic powders (i.e., powder D series), which means that the powder D series experiences the processing history of grinding → annealing → regrinding.

The structure information on the as-sintered ceramic bulk was also collected by neutron powder diffraction (ND) technology, since the neutron beam can penetrate the ceramic bulk. The ND data were collected at a wavelength of λ ∼ 1.63 Å using WOMBAT,^70^ a high-intensity powder diffractometer at the Australian Centre for Neutron Scattering, Australian Nuclear Science and Technology Organization. The pellet samples were attached to a sample stage and rotated in 15° increments around the vertical axis. Thirteen patterns in total were averaged to obtain the NPD pattern for further refinement analysis.^9^ The refinement with selected ceramic bulk is carried out using the Fullprof suite program.^71^

The differential scanning calorimetry (DSC) analysis of ground ceramic powders was also performed using a Mettler Toledo DSC-822 (Mettler, Toledo) on both heating and cooling cycles. For characterizing electrical properties, the polished ceramic bulk samples were coated with an Au electrode. The polarization–field (P–E) hysteresis loops were obtained using a ferroelectric hysteresis measurement tester (aixACCT TFanalyzer 2000).

## 3 Results and Discussions

To know the entire structure characteristic of the as-sintered ceramic bulk, Figure 1 shows the refined neutron diffraction (ND) patterns of the as-sintered ANLT4.5, ANLT5.3, and ANLT6 ceramic bulks, since the neutron beam can penetrate the entire ceramic bulk, which can present the entire structure information on them. The refinement parameters are listed in Tables S1 and S2. As shown in Figure 1, the structure of the ANLT4.5 ceramic bulk contains a nonpolar *Pbcm* phase. At higher ANLT compositions, the ceramic bulk contains a polar *R*3*c* phase with increasing Wt fraction, indicating that the investigated three chemical compositions are close to or in the AFE/FE phase boundary of the ANLT ceramic solid solution.

**Figure 1:**
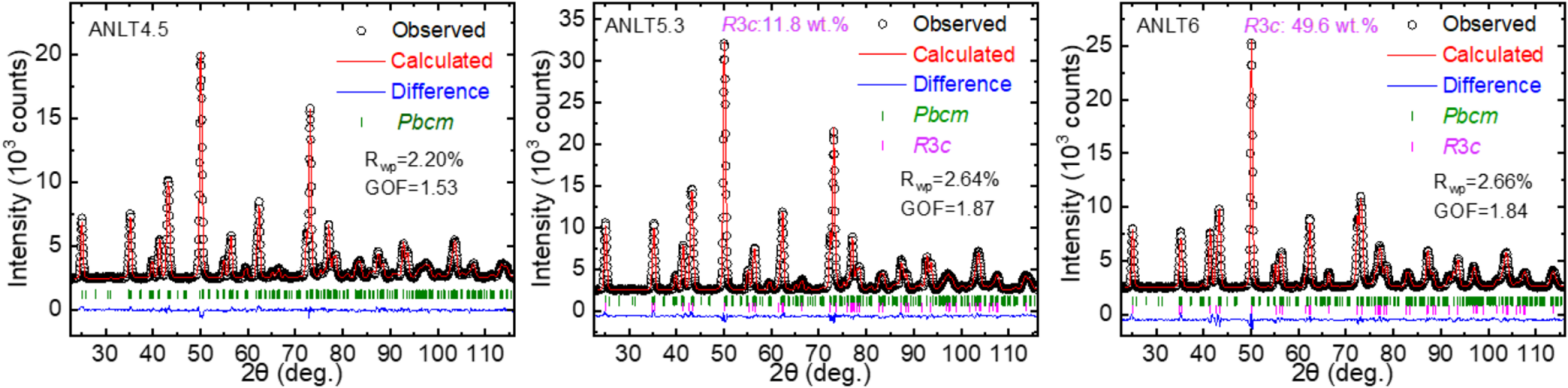
Refined neutron diffraction (ND) patterns of ANLT4.5, ANLT5.3, and ANLT6 ceramic bulks using nonpolar *Pbcm* and/or polar *R*3*c* space group models.

To elucidate the effects of mechanical and thermal treatments on the crystal structure of the as-sintered ANLT4.5, ANLT5.3, and ANLT6 ceramics, Figure 2 presents the selected (200)_p_, (211)_p_, and (220)_p_ diffraction peaks of the samples subjected to different processing histories. A close inspection of the splitting characteristics of these principal Bragg diffraction planes (200)_p_, (211)_p_, and (220)_p_ reveals that mechanical polishing exerts a pronounced influence on the crystal structure of all investigated compositions. This influence is manifested by the emergence of additional Bragg diffraction peaks or the disappearance of original ones when comparing the as-sintered ceramics (A) with the mechanically polished samples (B). As shown in Figure 2, for the ANLT4.5 and ANLT5.3 ceramics, extra diffraction peaks appear near 56.7° at the (211)_p_ plane (denoted as *), following mechanical polishing. In the case of ANLT6, substantial modifications are observed in the splitting profile of the (200)_p_ reflection, where several initial diffraction peaks at the (200)_p_, (211)_p_, and (220)_p_ planes vanish, accompanied by the appearance of new ones (denoted as *). Notably, these polishing-induced structural distortions are largely reversible upon subsequent heat treatment at 600 °C, as evidenced by the restoration of the original splitting characteristics and peak profiles of the (200)_p_, (211)_p_, and (220)_p_ planes. The close similarity between the XRD patterns of the as-sintered (A) and annealed (C) ceramics further confirms the recovery of the original crystalline structure after annealing.

**Figure 2:**
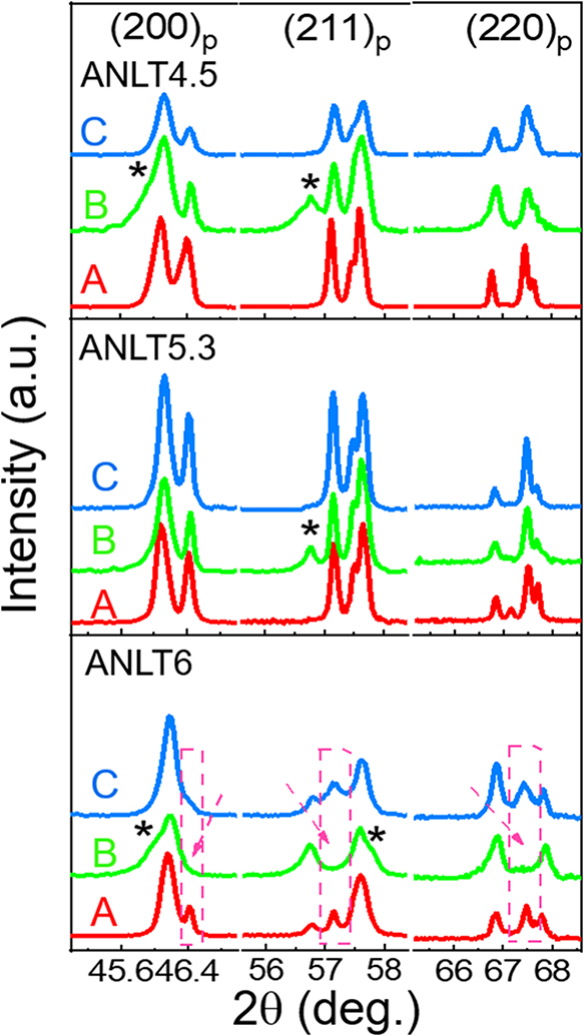
Expanded XRD patterns of ANLT4.5, ANLT5.3, and ANLT6 ceramic bulks, highlighting selected main reflections of the parent pseudocubic perovskite structure, (200)_p_, (211)_p_, and (220)_p_. The samples correspond to (A) as-sintered, (B) polished, and (C) annealed ceramic bulks.

To further clarify the influence of mechanical processing, the as-sintered bulks were crushed and ground (or milled) into fine ceramic powders, referred to as ground ceramic powders. During this process, most ceramic grains were subjected to mechanical forces, which can significantly modify their local structure. The ground powders were subsequently annealed at various temperatures (annealed ceramic powders), and the corresponding XRD patterns are presented in Figure S1. To verify the reproducibility of mechanically driven structural transitions, the powders annealed at 600 °C were lightly reground for a few seconds (reground ceramic powders). The local magnification of XRD data for these samples is shown in Figure 3a. As observed, the diffraction profiles of the (200)_p_, (211)_p_, and (220)_p_ reflections vary markedly depending on the processing history of the powders. To identify the phase transitions associated with grinding, annealing, and regrinding, Rietveld structure refinements were performed. The refined patterns, reliability factors, and lattice parameters are summarized in Figure S2 and Table S3, respectively. As shown in Figure 3b, the ANLT4.5 ground powders can be well described by the coexistence of *Pbcm* and *R3c* phases with weight fractions of 43.3 and 56.7%, respectively. After annealing at 600 °C, a single *Pbcm* phase is obtained, whereas a short regrinding treatment restores the coexistence of *Pbcm* (59.3 wt %) and *R3c* (40.7 wt %) phases, confirmed by improved refinement indicators (*R*_wp_ = 3.07%, χ^2^ = 2.28) compared with the single-phase model (*R*_wp_ = 4.81%, χ^2^ = 5.61) (Figure S3). Similar behavior is also observed in ANLT5.3. For ANLT6, the ground powders exhibit a pure *R3c* phase. After annealing at 600 °C, the structure transforms into a mixture of *Pbcm* and *R3c* phases, with *Pbcm* being dominant (65.4 wt %). A subsequent regrinding process reverses this ratio, resulting in *R3c* dominance (80.2 wt %). These findings unambiguously demonstrate that mechanical forces associated with grinding can induce an AFE-to-FE phase transition, while high-temperature annealing reverses this transition. Remarkably, even a brief manual regrinding of the annealed powders in an agate mortar for only a few seconds can again trigger the AFE-to-FE transition, highlighting the extreme sensitivity of the AFE phase to external mechanical perturbations.

**Figure 3:**
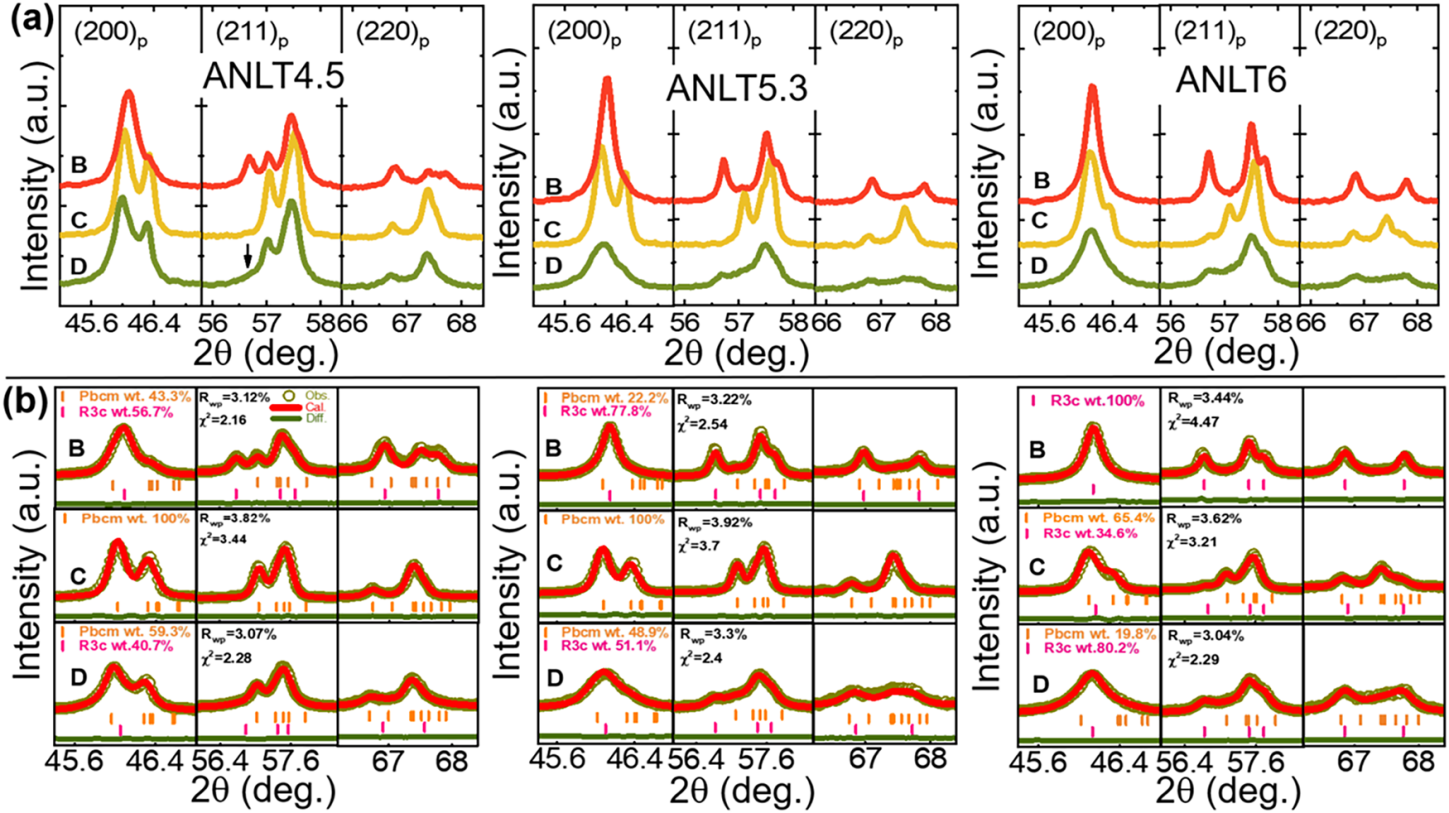
Expanded (a) raw XRD and (b) refined XRD patterns of ANLT4.5, ANLT5.3, and ANLT6 ceramic powders subjected to (B) grinding, (C) annealing, and (D) regrinding treatments. Selected main reflections of the parent pseudocubic perovskite structure, (200)_p_, (211)_p_, and (220)_p_, are highlighted.

A comparison of the structural configurations of the ceramic bulk and powders is summarized in Table 1. It is evident that for ANLT4.5, ANLT5.3, and ANLT6, the structures obtained after mechanical processing differ markedly from those of the bulk ceramics. Therefore, using the structures derived from mechanically processed powders to represent the intrinsic bulk structure may lead to misinterpretation of the structure–property relationships.

**Table 1:** Fraction of the R3c Phase in ANLT4.5, ANLT5.3, and ANLT6 Obtained from Different Samples Using Neutron (ND) Diffraction or XRD

		ceramic powders (XRD)		
composition	ceramic bulk (ND)	ground	annealed	reground
ANLT4.5	0	56.7 wt %	0	40.7 wt %
ANLT5.3	11.8 wt %	77.8 wt %	0	51.1 wt %
ANLT6	49.6 wt %	100 wt %	34.6 wt %	80.2 wt %

To further clarify whether mechanical treatment affects only the compositions located near or within the AFE/FE phase boundary, Figure 4a,b presents the expanded XRD patterns of the ground and annealed ceramic powders of the ANLT*x* solid solution, with compositions ranging from pure AN to ANLT6 (Figure S4). The structural configurations derived from the analyses are also indicated in the color bar in Figure 4a,b. As shown in Figure 4a,b, both the ground and annealed powders with compositions corresponding to AN, ANLT1.5, and ANLT3 exhibit identical splitting characteristics for the three principal perovskite diffraction planes, indicating that these compositions possess the same AFE *Pbcm* structure. This observation demonstrates that the AFE phase is structurally stable and is insensitive to mechanical processing. At higher *x* contents, specifically for compositions in the range of 3.8% ≤ *x* ≤ 5.3%, the ground powders exhibit mixed-phase diffraction features, indicative of the coexistence of AFE *Pbcm* and FE *R3c* phases. With further LT substitution (*x* = 6%), the structure transforms completely into a single FE *R3c* phase. For the annealed powders, compositions within 0 ≤ *x* ≤ 5.3% retain the characteristic splitting of the AFE *Pbcm* phase, while the ANLT6 composition exhibits the coexistence of AFE *Pbcm* and FE *R3c* phases. These results further confirm that compositions with *x* ≥ 3.8% are susceptible to mechanically induced phase transitions. The composition-dependent structural evolution in the ceramic bulks, determined using neutron diffraction (ND) measurements (Figure 4c), reveals a phase transition occurring at ANLT5.3, slightly different from that observed in the annealed powders (Figure 4b). This discrepancy suggests that residual stress in the sintered bulk may play a crucial role in stabilizing the FE *R3c* phase. A careful observation of the lattice parameter evolution of the AFE *Pbcm* phase with chemical compositions (see Figure S5) shows that the annealed ceramic powders show an evolutionary tendency similar to that of the ceramic bulk, which indicates a negligible change in chemical composition during crushing/grinding and annealing treatments. Moreover, although grinding the ceramic powders with chemical compositions of ANLT4.5 and ANLT5.3 does not completely transform the AFE *Pbcm* phase into FE *R*3*c* phase, the residual *Pbcm* lattice seems to be highly distorted (evidenced by the significant variation of lattice parameters when compared with annealed powders). The Williamson–Hall analysis results (see Figure S6 and Table S4) indicate that the differences in crystallite size (*S*) and microstrains (μ) in ANLT4.5 samples after different processing conditions are not very evident, in general, whereas a noticeable increase in microstrain is observed in both the *Pbcm* and *R*3*c* phases after regrinding the annealed ceramic powders. Notably, the microstrain associated with the FE *R*3*c* phase is consistently higher than that of the AFE *Pbcm* phase under the same processing conditions. These results suggest that lattice distortion plays a more prominent role in the structural evolution of ANLT4.5 induced by mechanical processing. The higher microstrain tolerance of the *R*3*c* phase implies that this phase is preferentially favored under mechanical stress through enhanced lattice distortion. Upon grinding, the *Pbcm* phase initially accommodates the applied stress, mainly via lattice distortion and microstrain accumulation without a detectable average symmetry change. With further strain accumulation, localized structural instability is induced, which facilitates partial transformation from the AFE *Pbcm* phase to FE *R*3*c*. A schematic summary of the composition-driven structural configuration and the structural evolution induced by mechanical and thermal treatments is illustrated in Figure 4d.

**Figure 4:**
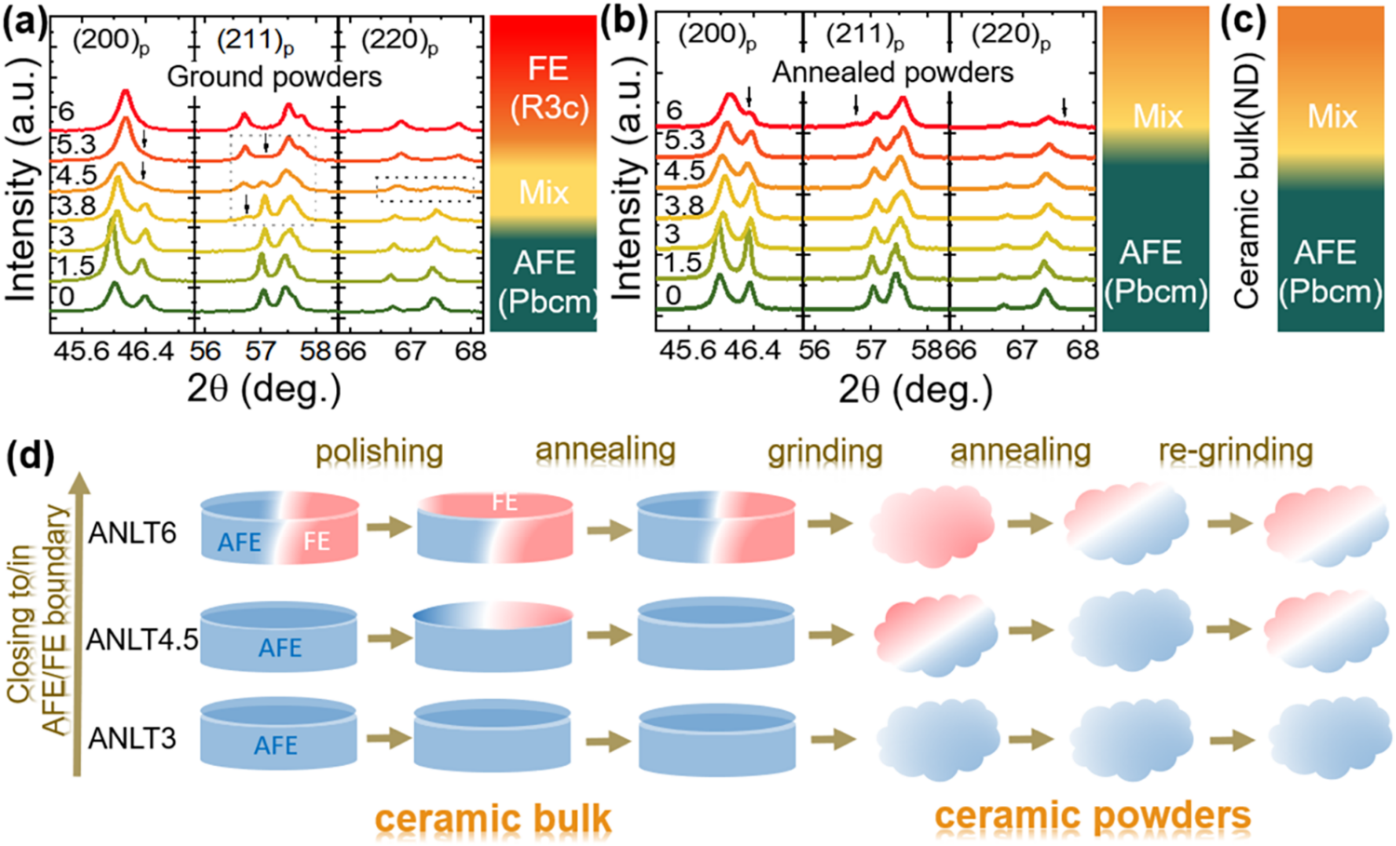
Expanded XRD patterns of ANLT*x* ceramic powders subjected to (a) grinding and (b) annealing. The composition-driven structural configurations for the ground and annealed powders, as well as for (c) ceramic bulks, are indicated by color bars. (d) Schematic illustration of phase transitions induced in ceramic bulks/powders by mechanical and heat treatments with compositions close to/in the AFE/FE phase boundary.

The classic stress-sensitive phase transitions have been extensively reported in Y-doped ZrO_2_ ceramics, where a thermodynamically favored monoclinic–tetragonal phase transition occurs near ∼1170 °C during heating and ∼950 °C during cooling.^72^ The low-temperature monoclinic phase is generally thermodynamically stable, while the high-temperature tetragonal phase can be retained at room temperature in a metastable state through Y doping. Once external stress is applied beyond a certain extent, these metastable tetragonal phases can irreversibly transform into the monoclinic phase. Figure 5a presents the DSC data of ANLT6 ground powders to examine whether a thermodynamically driven phase transition exists in the ANLT*x* system. As shown in Figure 5a, upon a heating–cooling cycle, sharp endothermic/exothermic peaks are observed around ∼420 and ∼370 °C for ANLT4.5, accompanied by a pronounced thermal hysteresis, indicating a first-order phase transition associated with the AFE–paraelectric (PE) transition.^36^ This transition shifts to higher temperatures for ANLT5.3 and ANLT6. Furthermore, no significant thermal events are detected below this first-order phase transition. However, careful inspection reveals that a deviation in the baseline slope occurred around ∼60 °C for ANLT4.5, ∼120 °C for ANLT5.3, and ∼140 °C for ANLT6 in the heating data. A similar deviation is also observed in the cooling curves, although at slightly lower temperatures. By magnifying the deviated baseline regions in the heating curves (see the inset), we can observe a broad endothermic feature for ANLT4.5, which becomes sharper at ANLT5.3 and more pronounced at ANLT6. This endothermic feature indicates a thermodynamically driven phase transition upon heating, while no corresponding exothermic event is detected during cooling within the same temperature range. These results suggest that heating removes the stress-stabilized FE (*R*3*c*) phase fraction and/or reduces the originally existing *R*3*c* phase fraction, resulting in the observed endothermic response. In other words, the stress-induced and/or preexisting FE (*R*3*c*) phase disappears at approximately 60, 120, and 140 °C for ANLT4.5, ANLT5.3, and ANLT6, respectively.

**Figure 5:**
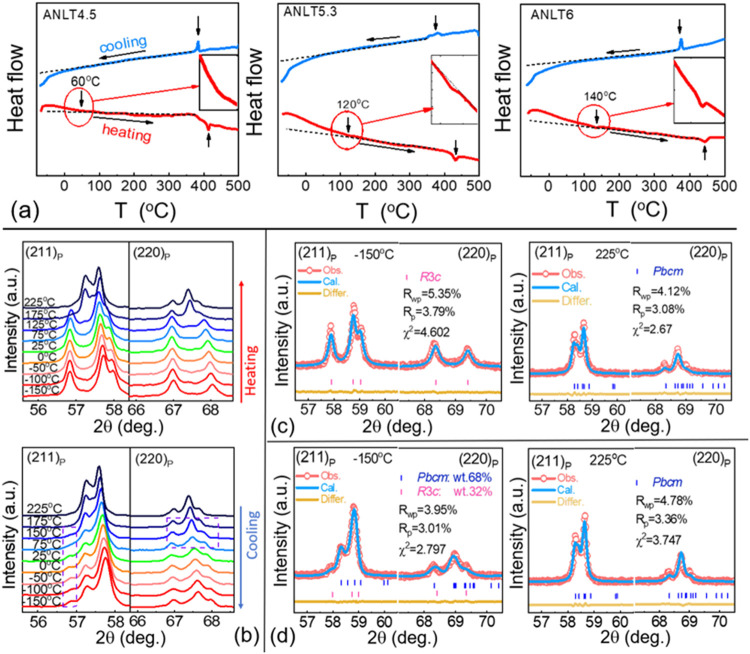
(a) DSC curves of ANLT4.5, ANLT5.3, and ANLT6 ground ceramic powders during a heating → cooling cycle. (b) *In situ* expanded XRD patterns of ANLT6 ground ceramic powders during heating and cooling, highlighting the (211)_p_ and (220)_p_ reflections of the parent pseudocubic perovskite structure. Magnified views of the refined XRD patterns of ANLT6 ground powders at the selected (211)_p_ and (220)_p_ reflections: (c) during heating at −150 and 225 °C and (d) during cooling at −150 and 225 °C.

To confirm the stress-induced AFE (*Pbcm*) → FE (*R*3*c*) phase transition associated with the thermodynamically driven transformation, *in situ* XRD measurements were conducted on ground ANLT6 ceramic powders during heating and cooling cycles, as a representative case. The raw *in situ* XRD patterns are presented in Figure S7, and the magnified views of the perovskite (211)_p_ and (220)_p_ reflections are summarized in Figure 5b. As shown in Figure 5b, the splitting characteristics of the (211)_p_ and (220)_p_ diffraction planes remain unchanged when the ground ceramic powders are heated from −150 to 125 °C. Upon further heating to higher temperatures (175 and 225 °C), the splitting features change significantly, indicating the occurrence of a phase transition between 125 and 175 °C. When cooling from 225 °C, the splitting characteristics of the (211)_p_ and (220)_p_ reflections are identical to those observed at 225 °C during heating. However, as the temperature decreases to 75 °C, the diffraction peak profiles of both reflections show slight variations. In particular, an additional splitting peak appears at 2θ ≈ 56.8°, which becomes more pronounced upon further cooling to −150 °C (Figure 5b). Structural refinement of the XRD data during heating, shown in Figure 5c, indicates a temperature-driven FE (*R*3*c*) → AFE (*Pbcm*) phase transition, consistent with the sharp endothermic event observed at ∼140 °C in the DSC curve (see Figure 5a). In contrast, structural analysis of the cooling data reveals that while the structure at 225 °C remains AFE (*Pbcm*), the configuration at −150 °C consists of coexisting AFE (*Pbcm*) and FE (*R*3*c*) phases, with the AFE (*Pbcm*) phase being dominant (≈ 68 wt %). This explains the absence of any sharp exothermic peaks in the DSC data during cooling. Notably, during cooling from 125 to −150 °C, the refined fraction of the *R*3*c* phase remains nearly constant (≈32–35 wt %, see Figure S8), which is lower than that in the ceramic bulk (≈49.6 wt %, see Table 1), indicating that the *Pbcm* and *R*3*c* phases coexist in a thermodynamic equilibrium. Based on the combined *in situ* XRD and DSC results, it can be concluded that heating the ground ANLT6 powders to high temperatures causes both the stress-induced and the originally existing FE (*R*3*c*) phases to transform into the AFE (*Pbcm*) phase as the internal stress is released. Upon subsequent cooling, most of the powders retain the AFE (*Pbcm*) phase in a metastable state due to the absence of stress, leading to no obvious exothermic event in the DSC curve. When these metastable AFE (*Pbcm*) powders are subjected to mechanical grinding, the stress-induced AFE (*Pbcm*) → FE (*R*3*c*) phase transition occurs again.

To further clarify that the stress-sensitive AFE (*Pbcm*) → FE (*R*3*c*) transition is only intimately related to the existence of a metastable AFE phase, Figure 6 presents both the composition- and temperature-dependent ferroelectric (P–E) loops of ANLT*x* ceramic bulks. As shown in Figure 6a, compositions with *x* ≤ 3.8% exhibit classic double-polarization hysteresis loops accompanied by gradually decreased *E*_F_/*E*_A_, indicating a progressive reduction in the stability of the AFE phase. For these compositions, the AFE phase can recover either after removing the electric field (as in AN and ANLT3) or by applying a small reverse field (as in ANLT3.8).

**Figure 6:**
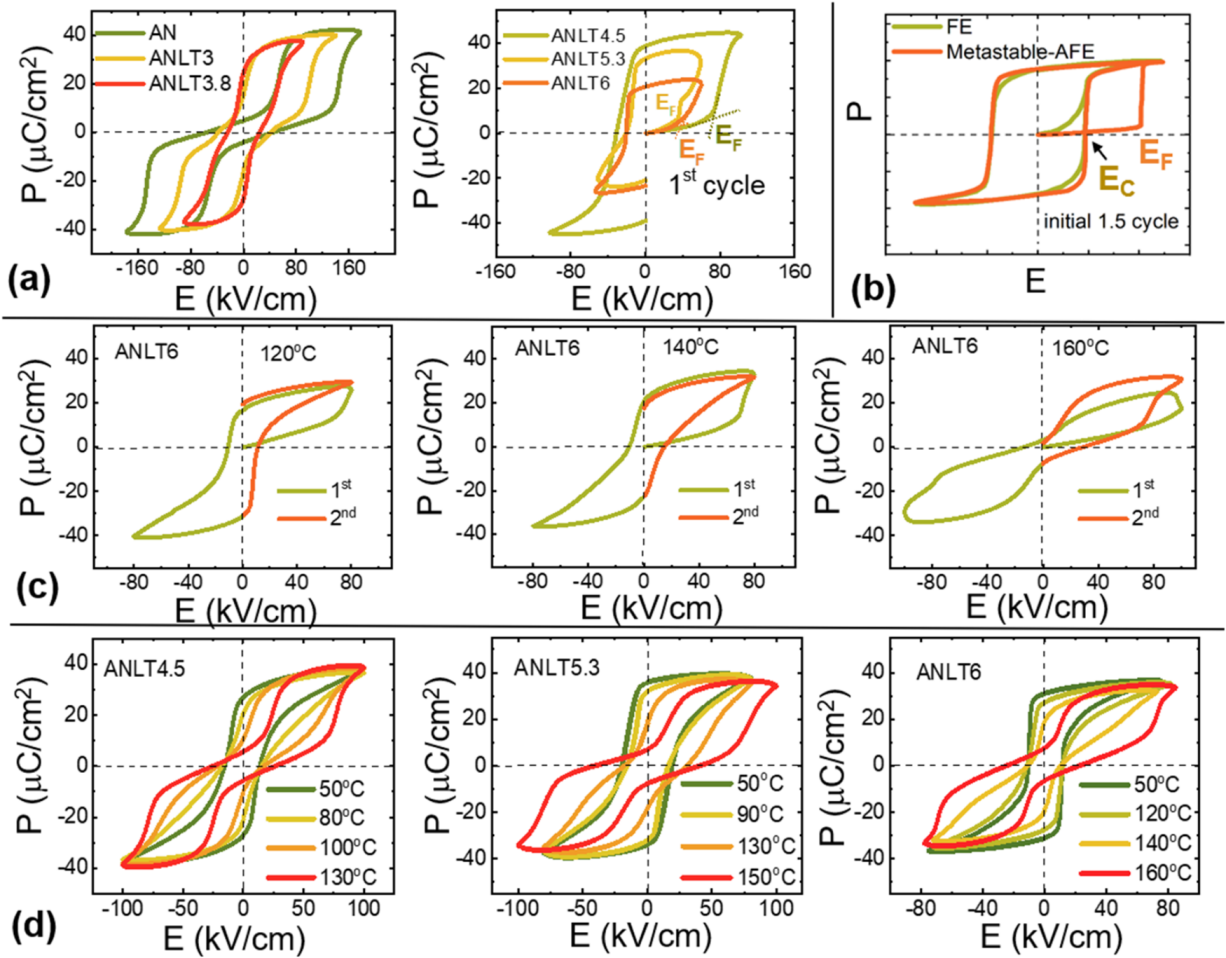
(a) Polarization–electric field (P–E) hysteresis loops of ANLTx ceramic bulks. (b) Schematic illustration of the initial 1.5-cycle P–E loops for a typical FE phase and a metastable AFE phase. (c) Temperature-driven initial 1.5-cycle P–E loops of ANLT6 ceramic bulk. (d) Temperature-driven successive P–E loops of ANLT4.5, ANLT5.3, and ANLT6 ceramic bulks. Note: *E*_F_ denotes the critical electric-field-induced AFE → FE phase transition; *E*_C_ denotes the coercive field for the polarization reversal of the FE phase.

When the composition approaches or enters the AFE/FE boundary region (i.e., ANLT4.5, ANLT5.3, and ANLT6), the AFE phase becomes metastable (Figure 6b),^3^ as the structure transforms into the FE phase after removing the electric field and cannot revert to the original AFE phase even under a reverse field. It is noteworthy that the initial (first cycle) P–E loop of the ANLT6 ceramic bulk exhibits mixed features of both typical FE and metastable AFE behaviors, further confirming that this composition contains coexisting AFE and FE phases, consistent with the structural configuration of the ceramic bulk (see Table 1). The AFE (*Pbcm*) → FE (*R*3*c*) transition induced by an electric field has been confirmed in our previous study,^58^ where a continuously increased fraction of the FE *R*3*c* phase was observed under repeated low-field cycling, highlighting the extreme sensitivity of the AFE *Pbcm* phase to external electric field stimuli. For the ANLT6 ceramic bulk, when heated from 140 to 160 °C (Figure 6c), the P–E loop characteristic of the metastable AFE phase transforms into a double-polarization hysteresis loop corresponding to a more stable AFE phase, suggesting that below 160 °C, the remaining AFE phase in the ceramic bulk is metastable. Furthermore, the temperature-dependent cyclic P–E loops of ANLT4.5, ANLT5.3, and ANLT6 ceramics (see Figure 6d) reveal that the electric-field-induced and/or originally existing FE phases revert to a more stable AFE phase at 80, 120, and 140 °C, respectively. These transition temperatures are in good agreement with the thermal events observed in the DSC data and the phase transitions detected by *in situ* XRD. These results further confirm the metastable nature of the AFE phase below the transition temperature and demonstrate its extreme sensitivity to external stimuli, including both electric fields and mechanical stress. The compositions and temperature ranges corresponding to the existence of the metastable AFE phase are summarized in Figure 7. We believe that these findings not only provide a more precise understanding of the structure–property relationships in this ceramic system but also offer guidance for designing electroceramics (and their composites) with specific electrical and mechanical performance through the controlled manipulation of residual stress.

**Figure 7:**
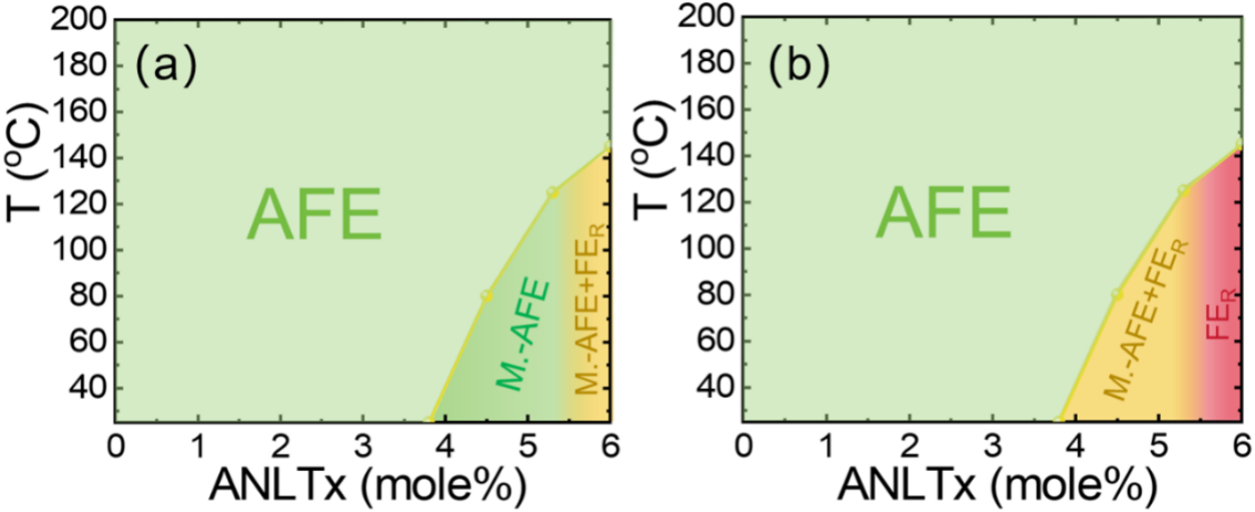
Proposed local temperature–composition phase diagram of the ANLT*x* solid solution: (a) ceramic bulk and (b) ground ceramic powders (note: M.-AFE: metastable antiferroelectric; FE_R_: FE rhombohedral phase).

The metastable AFE phase, characterized by irreversible AFE–FE hysteresis loops (see Figure 6b), has been extensively investigated over the past decades, particularly in Pb-based AFE systems, due to its excellent electrostrain and electromechanical performance. Our study reveals that this metastable AFE phase is extremely sensitive not only to electric fields, the most widely studied external stimulus, but also to the stress generated during mechanical processing. Manual polishing/grinding of the ceramic bulks/powders introduces shear forces, which can generate local shear strain, accompanied by microscopic compressive strain. These strains can modify the tilting of oxygen octahedra as well as the off-centering of cations; in other words, shear stress can couple with spontaneous polarization and the tilting modes of oxygen octahedra. Such effects can further reduce the energy barrier for the *Pbcm* → *R*3*c* transition, facilitating nucleation and growth of the FE *R*3*c* phase in a martensitic or twinning-like manner. Recent studies on NaNbO_3_, a classic lead-free metastable AFE prototype, have directly demonstrated that the combination of residual shear and compressive strain can trigger the nucleation of an FE phase.^73^ Notably, a similar mechanism has been suggested in a recent study of hand-milling-induced phase transitions in marcasite-type carbodiimides (Ba_0.9_M_0.1_NCN, M = Ca, Sr), where shear forces induce sliding of Ba^2+^ cations and rotation of NCN^2–^ anions, leading to a stress-induced marcasite-type orthorhombic → CsCl-type tetragonal transition. These previous studies strongly support our deduction regarding the stress-induced AFE → FE transition in ANLT*x* ceramics.^74^

## 4 Conclusions

In summary, we investigated the (1–*x*)­AgNbO_3_–*x*LiTaO_3_ (ANLT*x*, *x* = 4.5, 5.3, and 6 mol %) ceramic solid solutions as a representative system to examine the effects of essential processing procedures, including mechanical treatments (i.e., polishing and grinding) and heat treatments, on the structure of both ceramic bulks and powders. It was found that compositions near or within the composition-driven antiferroelectric (AFE)/ferroelectric (FE) phase boundary are highly sensitive to their processing history. Mechanical forces applied during polishing or grinding can induce an AFE (*Pbcm*) → FE (*R*3*c*) phase transition, which can be reversed through high-temperature annealing. Further investigations revealed that the mechanically induced FE *R*3*c* phase is closely linked to a thermodynamically driven phase transition associated with the metastable AFE phase. These findings provide new insights into AFE/FE phase transitions, emphasizing that the effects of processing history must be carefully considered to comprehensively understand the structure–property relationships and to control the behavior of this class of AFE materials.

## Supplementary Material



## Notes

XRD raw data, refined patterns, lattice parameters, reliable factors, and the Williamson–Hall plot (PDF)
