# Supplementary material for: Susceptible Antiferroelectric/Ferroelectric Transitions in Silver Niobate-Based Ceramics Induced by Manual Mechanical Processing
Source: Inorg Chem. 2026 Feb 4;65(6):3215–24. doi: 10.1021/acs.inorgchem.5c03344 (PMC12914626; doi:10.1021/acs.inorgchem.5c03344)
Supplement: Supplementary file 1 [file ic5c03344_si_001.pdf]

Supporting Information

Susceptible Antiferroelectric/Ferroelectric Transitions in Silver Niobate-Based Ceramics Induced by Manual Mechanical Processing

Ye Tian[a,b,c*#], Teng Lu[c#], Shaoqi Guo[a], Chen Chen[d], Liaona She[e], Li Jin[b], Ray L. Withers[c], Yuanting Wu[a], Wanyin Ge[a], Xiaoyong Wei[b], Haixue Yan[f*] and Yun Liu[c*]

[a]School of Materials Science and Engineering, Shaanxi University of Science & Technology, Xi'an,710021, Shaanxi Province, P.R. China

[b]Electronic Materials Research Laboratory, School of Electronic and Information Engineering, Xi'an Jiaotong University, Xian, 710049, China.

[c]Research School of Chemistry, The Australian National University, ACT 2601, Australia

[d]State Key Laboratory of High Performance Ceramics and Superfine Microstructure, Shanghai Institute of Ceramics Chinese Academy of Sciences, Shanghai, 200050, China

[e]Institute of Science and Technology for New Energy, Xi'an Technological University, Xi'an, 710021 P. R. China

[f]School of Engineering and Materials Science, Queen Mary University of London, Mile End Road, London E1 4NS, UK.

[#]Author contributions: Ye Tian and Teng Lu contributed equally to this work.

*The corresponding author.

E-mail: ye.tian@sust.edu.cn, h.x.yan@qmul.ac.uk and yun.liu@anu.edu.au

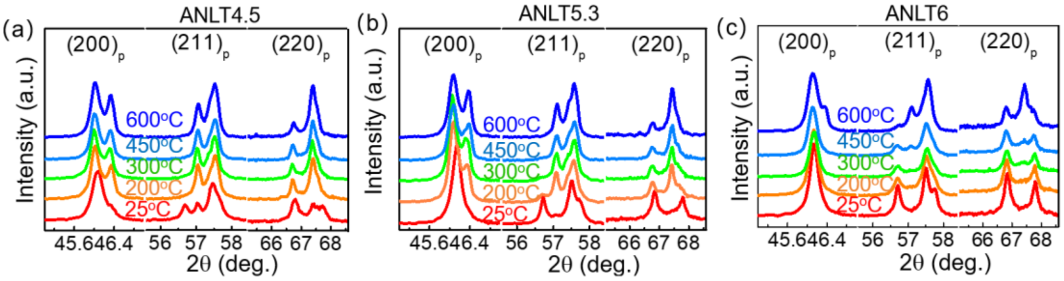

Figure.S1 Expanded XRD patterns of heat-treated (a) ANLT4.5, (b) ANLT5.3, and (c) ANLT6 ceramic powders selected main $(200)_p/(211)_p/(220)_p$ diffraction reflections of parent pseudo-cubic perovskite structure (Note: the XRD pattern at 25°C is the data of ground ceramic powders.)

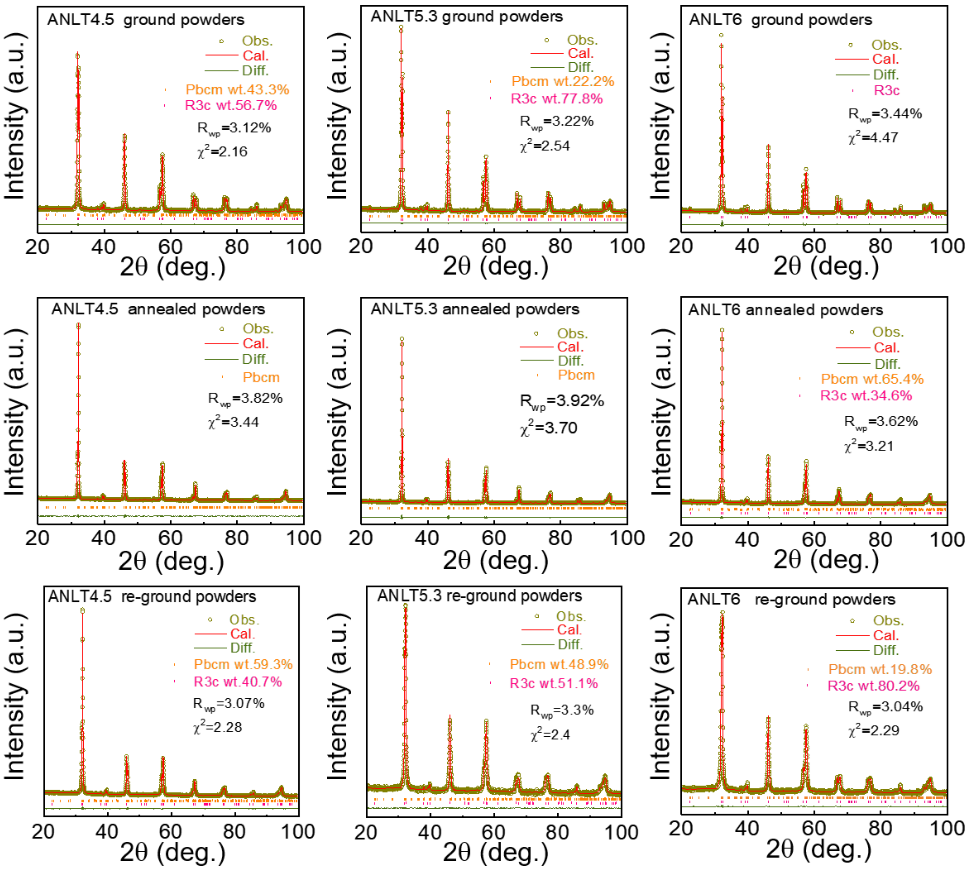

Figure S2. Refined XRD patterns of ground, annealed and re-ground ceramic powders of ANLT4.5, ANLT5.3, and ANLT6(using nonpolar *Pbcm* and polar *R*3*c* structure models).

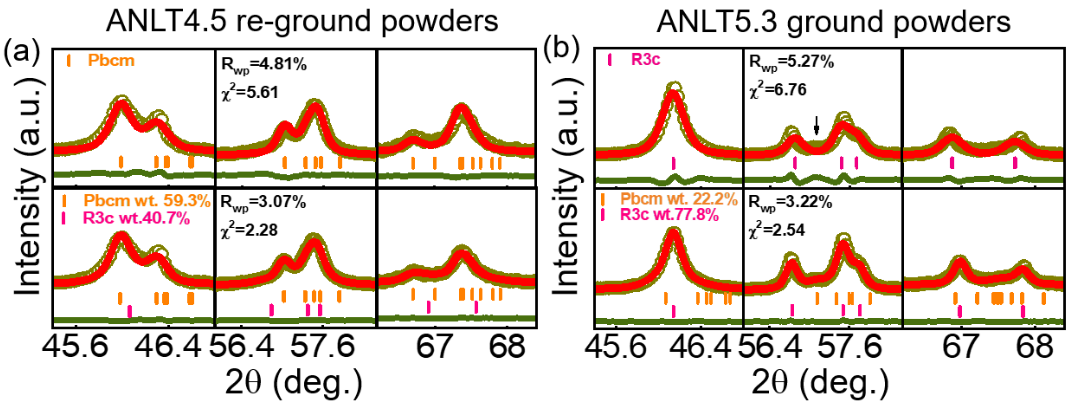

Figure S3. Expanded Refined-XRD patterns of (a) ANLT4.5 ground powders and (b) ANLT5.3 re-ground powders using single *R*3*c*/*Pbcm* phase or two phases' models.

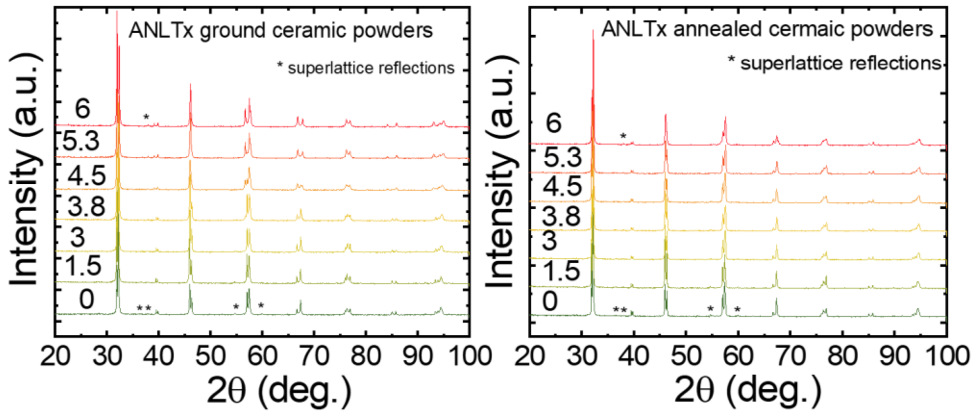

Figure.S4 Raw XRD data of ANLT*x* ground and annealed ceramic powders

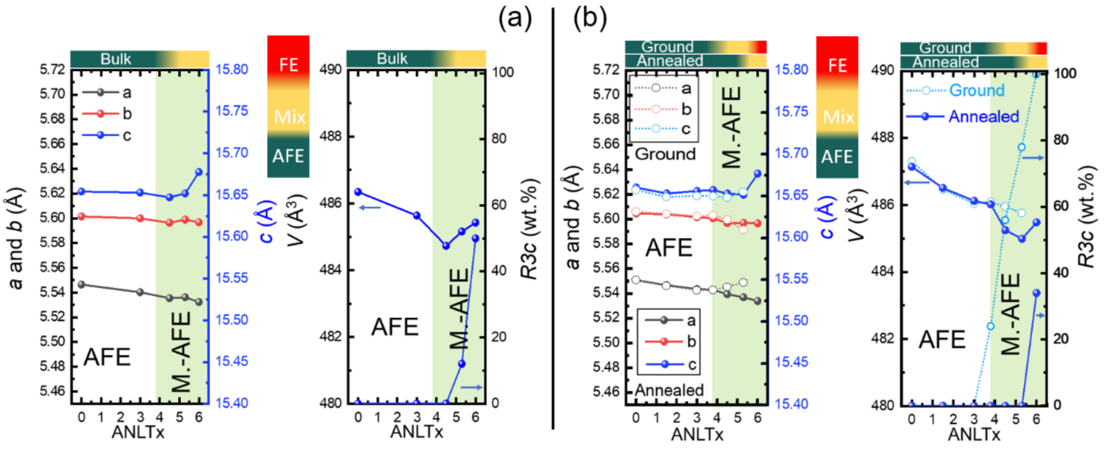

Figure. S5 AFE *Pbcm* lattice parameters (*a*, *b*, *c* and *V*) and *R*3*c* phase fraction(wt.%) as a function of ANLTx: (a) ceramic bulk (ND data) and (b) ground/annealed ceramic powders (XRD data)(color bar, dark green: AFE *Pbcm*; yellow: *Pbcm*+*R*3*c*; Red: FE *R*3*c*)

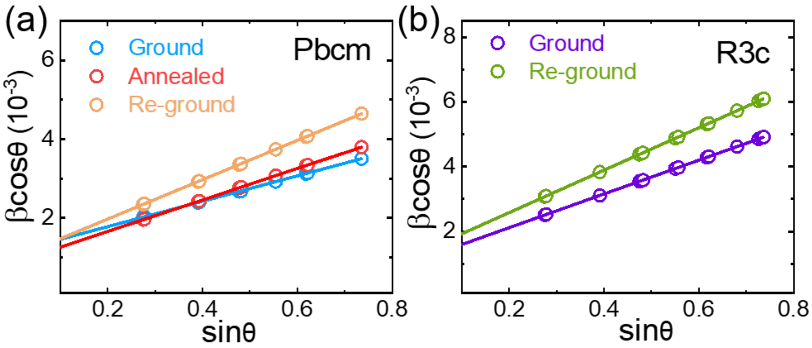

Figure. S6 Willamson-Hall plots of the (a) *Pbcm* and/or (b) *R3c* phases in ground, annealed and re-ground ANLT4.5 ceramic powders.

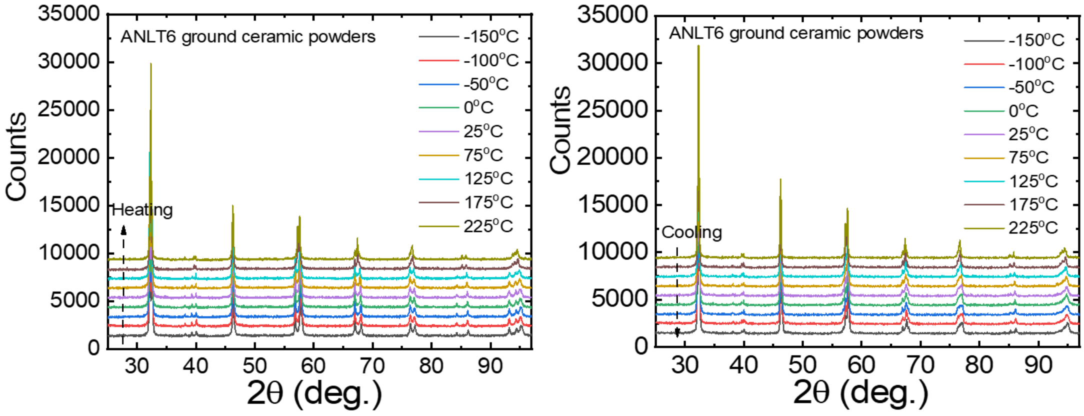

Figure.S7 *in situ* high-temperature XRD patterns of ground ANLT6 ceramic powders with a heating and cooling cycle.

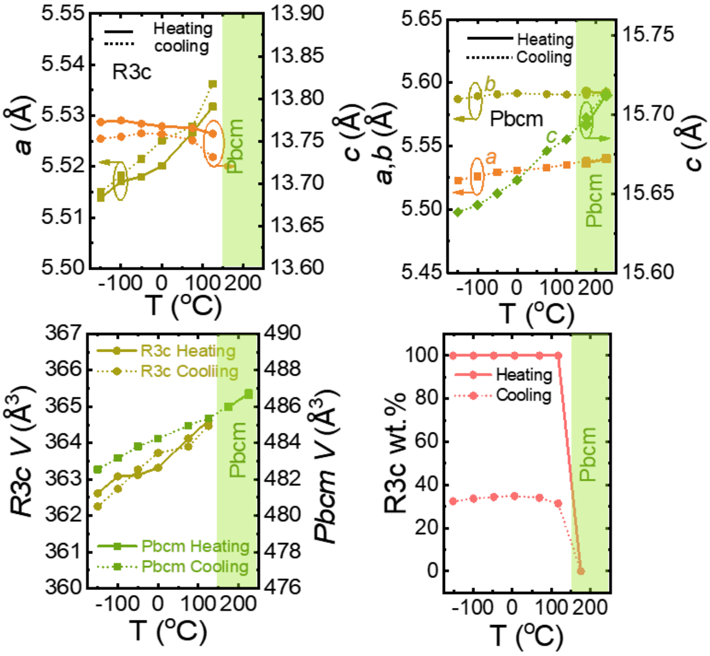

Figure S8. Refined lattice parameters and phase fraction as a function of temperature of ANLT6

ground ceramic powders with a heating and cooling cycle. (light green: *Pbcm*)

Table S1 Refinement details and refined structural parameters for ANLT4.5, ANLT5.3 and ANLT6 ceramic bulk using non-polar *Pbcm* and/or polar *R*3*c* space groups structural models.

Chemical composition		ANLT4.5	ANLT5.3		ANLT6	
Space group		*Pbcm*	*Pbcm*	*R3c*	*Pbcm*	*R3c*
Unit-cell parameters	*a*	5.5356 (8)	5.5363(8)	5.5241(7)	5.5318(6)	5.5244(6)
	b	5.5961(8)	5.5985(8)	5.5241(7)	5.5972(8)	5.5244(6)
	c	15.6475(15)	15.6538(12)	13.7957(9)	15.6782(11)	13.7856(8)
R_{wp}		2.20%	2.64%	2.64%	2.66%	2.66%
RF		2.17%	2.32%	1.82%	2.65%	1.45%
GOF		1.53	1.87	1.87	1.84	1.84
Phase Fraction(wt.%)		–	88.2%	11.8%	`50.4%	49.6%

Table S2 Atomic position for ANLT4.5, ANLT5.3 and ANLT6 ceramic bulk using non-polar *Pbcm* and/or polar *R*3*c* space groups structural models.

	Space group	*Pbcm*	Fractional coordinate			Uiso(Å^2)
	Atomic label	Wyckoff positions	x	y	z	
ANLT4.5	Ag1/Li1	4c	0.752(2)	0.25	0	0.015(1)
	Ag2/Li2	4d	0.256(2)	0.765(1)	0.25	0.015(1)
	Nb1/Ta1	8e	0.755(1)	0.730(2)	0.8741(6)	0.008(1)
	O1	4c	0.3103(26)	0.25	0	0.013(2)
	O2	4d	0.8052(26)	0.7308(11)	0.25	0.013(2)
	O3	8e	-0.0333(8)	0.0394(8)	0.889(1)	0.013(2)
	O4	8e	0.5278(7)	0.4773(10)	0.861(1)	0.013(2)
	Space group	*Pbcm*	Fractional coordinate			Uiso(Å^2)
	Atomic label	Wyckoff positions	x	y	z	
ANLT5.3	Ag1/Li1	4c	0.7485(20)	0.25	0	0.015(1)
	Ag2/Li2	4d	0.2657(17)	0.7639(21)	0.25	0.015(1)
	Nb1/Ta1	8e	0.7543(13)	0.7299(23)	0.8726(8)	0.011(1)
	O1	4c	0.3185(14)	0.25	0	0.015(2)
	O2	4d	0.7976(16)	0.7342(10)	0.25	0.015(2)

	O3	8e	-0.0313(7)	0.0352(8)	0.8879(9)	0.015(2)
	O4	8e	0.5280(7)	0.4755(9)	0.8601(9)	0.015(2)
	Space group	*R*3*c*	Fractional coordinate			Uiso(Å^2)
	Atomic label	Wickoff site	x	y	z	
	Ag1/Li1	6a	0	0	0.3268(15)	0.015(1)
	Nb1/Ta1	6a	0	0	0.0798(10)	0.011(2)
	O1	18b	0.5737(19)	0.0273(19)	0.3165(8)	0.015(2)
ANLT6	Space group	*Pbcm*	Fractional coordinate			Uiso(Å^2)
	Atomic label	Wickoff site	x	y	z	
	Ag1/Li1	4c	0.7463(21)	0.25	0	0.012(1)
	Ag2/Li2	4d	0.2558(22)	0.7759(19)	0.25	0.012(1)
	Nb1/Ta1	8e	0.7492(23)	0.7527(18)	0.8737(8)	0.009(1)
	O1	4c	0.3240(22)	0.25	0	0.012(2)
	O2	4d	0.7823(23)	0.7300(29)	0.25	0.012(2)
	O3	8e	-0.0245(15)	0.0381(15)	0.8889(8)	0.012(2)
	O4	8e	0.5137(16)	0.4954(30)	0.8591(7)	0.012(2)
	Space group	*R*3*c*	Fractional coordinate			Uiso(Å^2)
	Atomic label	Wickoff site	x	y	z	
	Ag1/Li1	6a	0	0	0.3246(6)	0.012(1)
	Nb1/Ta1	6a	0	0	0.0819(8)	0.009(2)
	O1	18b	0.5677(12)	0.0098(11)	0.3165(8)	0.012(2)

Table S3 Refinement details and refined structural parameters for ground, annealed and re-ground ANLT4.5, ANLT5.3, ANLT6 ceramic powders using non-polar *Pbcm* and/or polar *R*3*c* space groups structural models.

Composition		$(Ag_{0.955}Li_{0.045})(Nb_{0.955}Ta_{0.045})O_3$				
Processing history		Ground		Annealed	Re-ground	
Phase configuration		*Pbcm*	*R*3*c*	*Pbcm*	*Pbcm*	*R*3*c*
Unicell parameters	*a*	5.5455(4)	5.5298(3)	5.53921	5.5384(9)	5.5387(9)
	b	5.5998(1)	5.5298(3)	5.59672	5.5996(0)	5.5387(9)
	c	15.6486(6)	13.7822(0)	15.65248	15.6485(6)	13.7375(4)
	V	485.95	364.98	485.24	485.31	364.98
Phase fraction(wt.)		43.3%	56.7%	-	59.3%	40.7%
R_{wp}		3.12%		3.82%	3.07%	
GOF(χ^2)		2.16		3.44	2.28	
Composition		$(Ag_{0.947}Li_{0.053})(Nb_{0.947}Ta_{0.053})O_3$				
Processing history		Ground		Annealed	Re-ground	
Phase configuration		*Pbcm*	*R*3*c*	*Pbcm*	*Pbcm*	*R*3*c*
Unicell parameters	*a*	5.5491(4)	5.5262(7)	5.5366(7)	5.5395(1)	5.5260(3)
	b	5.5916(7)	5.5262(7)	5.5967(4)	5.5954(5)	5.5260(3)
	c	15.6551(2)	13.7877(6)	15.6512(1)	15.6490 (3)	13.7722(0)
	V	485.76	364.66	484.99	485.06	364.22

Phase fraction(wt.)		22.2%	77.8%	-	48.9%	51.1%
R_{wp}		3.22%		3.92%	3.3%	
GOF(χ^2)		2.54		3.70	2.40	

Composition		$(Ag_{0.94}Li_{0.06})(Nb_{0.94}Ta_{0.06})O_3$				
Processing history		Ground	Annealed		Re-ground	
Phase configuration		*R*3*c*	*Pbcm*	*R*3*c*	*Pbcm*	*R*3*c*
Unicell parameters	*a*	5.5261 (8)	5.5337(5)	5.5317(7)	5.5447(4)	5.5253(7)
	b	5.5261(8)	5.5963(8)	5.5317(7)	5.5943(5)	5.5253(7)
	c	13.7825(2)	15.6763(7)	13.7635(7)	15.6382(3)	13.7796(8)
	V	364.51	485.48	364.75	485.43	364.33
Phase fraction(wt.)		-	65.4%	34.6%	19.8%	80.2%
R_{wp}		3.44%	3.62%		3.04%	
GOF(χ^2)		4.47	3.21		2.29	

Table S4 Summary of the crystallite size and microstrain deduced from the Willamson-Hall plots of ANLT4.5 ceramic powders sample after different processing conditions.

	Pbcm			*R*3*c*	
	Ground	Annealed	Re-ground	Ground	Re-ground
S (nm)	135	179	158	144	121
μ (10^{-3})	0.80	1.00	1.25	1.30	1.63
